# Peptide microarray-based identification of dormancy-associated *Mycobacterium tuberculosis* antigens inducing immune responses among latent tuberculosis infection individuals in Thailand

**DOI:** 10.1038/s41598-023-34307-4

**Published:** 2023-04-28

**Authors:** Jariya Hanthamrongwit, Panicha Aruvornlop, Chutiphon Saelee, Nattiya Wanta, Passarun Poneksawat, Phyu Thwe Soe, Soe Paing Kyaw, Prasong Khaenam, Saradee Warit, Davide Valentini, Surakameth Mahasirimongkol, Panadda Dhepakson, Sakulrat Soonthornchartrawat, Patchanee Chootong, Chaniya Leepiyasakulchai

**Affiliations:** 1 Department of Clinical Microbiology and Applied Technology, Faculty of Medical Technology, Mahidol University, Bangkok, 10700 Thailand; 2 Center of Research and Innovation, Faculty of Medical Technology, Mahidol University, Bangkok, 10700 Thailand; 3 Department of Medical Laboratory Technology, University of Medical Technology, Mandalay, 05071 Myanmar; 4 Clinical Pathology Laboratory, (1000) Bedded General Hospital, Nay Pyi Taw, 15011 Myanmar; 5 Center of Standardization and Product Validation, Faculty of Medical Technology, Mahidol University, Bangkok, 10700 Thailand; 6 Industrial Tuberculosis Team, Industrial Medical Molecular Biotechnology Research Group, BIOTEC, National Science and Technology Development Agency, Thailand Science Park, Pathum Thani, 12120 Thailand; 7 Department of Laboratory Medicine, Karolinska Institutet, Stockholm, Sweden; 8 Department of Cellular Therapy and Allogeneic Stem Cell Transplantation (CAST), Karolinska University Hospital, Stockholm, Sweden; 9 Medical Life Sciences Institute, Department of Medical Sciences, Ministry of Public Health, Nonthaburi, Thailand

**Keywords:** Immunology, Microbiology, Medical research

## Abstract

Multi-stage tuberculosis (TB) vaccines composed of active- and dormancy-associated antigens are promising to trigger the immune protection against all TB stages. However, scientists are still in quest of the suitable vaccine candidates. In this study, we identified the potential targets for this vaccine in a high TB burden country, Thailand. Peptide microarray was applied to gauge IgA and IgG antibodies specific to 16,730 linear epitopes of 52 dormancy-associated *Mycobacterium tuberculosis* (*M. tb*) proteins in three study groups: active tuberculosis (ATB), latent tuberculosis infection (LTBI) and endemic healthy control (EHC). Preferential IgA recognition against epitopes of dormancy-associated proteins was identified in LTBI group. Validation of these findings revealed that LTBI subjects exhibited the greater levels of Rv2659c- and Rv1738-specific IgA than those of household contacts, but less than did ATB subjects. Frequencies of IFNγ-producing CD4^+^ and CD8^+^ T cells induced by proteins Rv2659c and Rv1738 were higher in LTBI than ATB individuals. The results indicated that LTBI group in a high TB burden country demonstrated cell-mediated immune response to proteins Rv2659c and Rv1738 stronger than those of ATB. These immune responses likely contribute to natural protection against dormant *M. tb* and might be potential targets for a multi-stage TB vaccine.

## Introduction

Tuberculosis (TB) caused by *Mycobacterium tuberculosis (M. tb)* continues to be a top-tier life-threatening disease globally. TB-elimination remains unachievable when a quarter of the world population harbours an *M. tb* reservoir, namely latent tuberculosis infection (LTBI)^1^. Specifically, LTBI individuals have a substantial risk (5–10% in their lifetime) of developing active tuberculosis (ATB), and thereby maintaining the incidence of new TB cases^2^. To tackle this problem, an idea based on prevention of TB reactivation among those with LTBI has been raised as a potential approach^3^. Until now, the World Health Organization (WHO) has recommended a vaccination strategy which enhances immune responses that suppress TB reactivation^4^. However, the only approved TB vaccine [Bacillus Calmette–Guérin (BCG)] does not confer effective protection against pulmonary TB, though it can moderate the severity of TB in children^5^. Therefore, development of a new and effective vaccine is required to efficiently control TB.

Among the new generation of vaccines, multi-stage vaccines composed of both active- and dormancy-phase antigens serve as the potential vaccines that provide immune protection against all TB stages^6^. Recent studies showed that vaccine candidates: ID93/GLA-SE and H56:IC31 reduce the bacterial burden and interfere with pulmonary pathogenesis in mice and non-human primates^7,8^. Clinical trial data also suggested that ID93/GLA-SE enhanced IFN-γ/TNF-α-producing CD4^+^ T cells in non-exposed TB persons, however only minimal Rv1813 (dormancy-associated antigen)-specific CD4^+^ T cells were detected^9^. H56:IC31 also induced robust Ag85B- specific CD4^+^ T cell responses among QuantiFERON-TB (QFT)-negative individuals; unfortunately, low frequency Rv2660c-specific CD4^+^ T-cell responses were observed in both QFT-negative and -positive individuals^10^. This suggests that active-phase antigens may be immunodominant, while the dormancy-associated antigens used in current vaccines are less immunogenic. Thus, ongoing investigations for new candidate dormancy-associated antigens are necessary.

During a latent *M. tb* infection, the immune-related structure called a ‘granuloma’ is formed which restrains *M. tb* multiplication within the toxic microenvironment^11^. This forces *M. tb* to persist as a non-replicating form in the focal aggregation of immune cells, by encoding over 65 dormancy-associated proteins that facilitate metabolic reduction and recovery from anaerobiosis^11,12^. The group of dormancy-associated proteins include DosR regulon-encoded proteins, universal stress proteins, starvation stimulon-encoded proteins and resuscitation-associated proteins^13,14^. Numerous studies have characterized the potential of dormancy-associated antigens as vaccine targets by examining natural immunity both in humoral and cellular immune responses. Most of them demonstrated antigen-specific IFN-γ response^15–17^ and revealed differences in the antibody profiles against those proteins among LTBI and ATB individuals^18,19^.

Despite the growing evidence of immune responses against dormancy *M. tb* antigens, immune profiles are reported to be distinct in different geographic regions. Hence, in this study, we used a peptide microarray technique to screen the linear B cell epitopes of 52 dormancy-associated *M. tb* antigens among LTBI, ATB and endemic healthy control study groups. We further investigated the humoral and cell mediated immune responses to recombinant Rv2659c (starvation stimulon-encoded protein), Rv1738 (DosR regulon-encoded protein) and early secreted antigenic target 6 kDa (ESAT-6) proteins in a Thai population. This is the first report from Thailand, a high TB burden country which uses BCG vaccination, to identify dormancy-associated *M. tb* targets for potential use in development of new multi-stage tuberculosis vaccines.

## Results

### Peptide specificity was more pronounced in IgA responses

Since not all LTBI individuals develop active tuberculosis, we anticipated that the high magnitude of antibody response against dormancy-associated *M. tb* antigens among LTBI individuals could help prevent the progression of active tuberculosis in addition to the cell-mediated immune compartment and those antigens could be the potential vaccine targets. Since there are abundant pool of dormancy-associated *M.tb* antigens, we utilized the benefit of peptide microarray approach to identify dormancy antigens that exclusively produce high responses among LTBI individuals.

**Figure 1:**
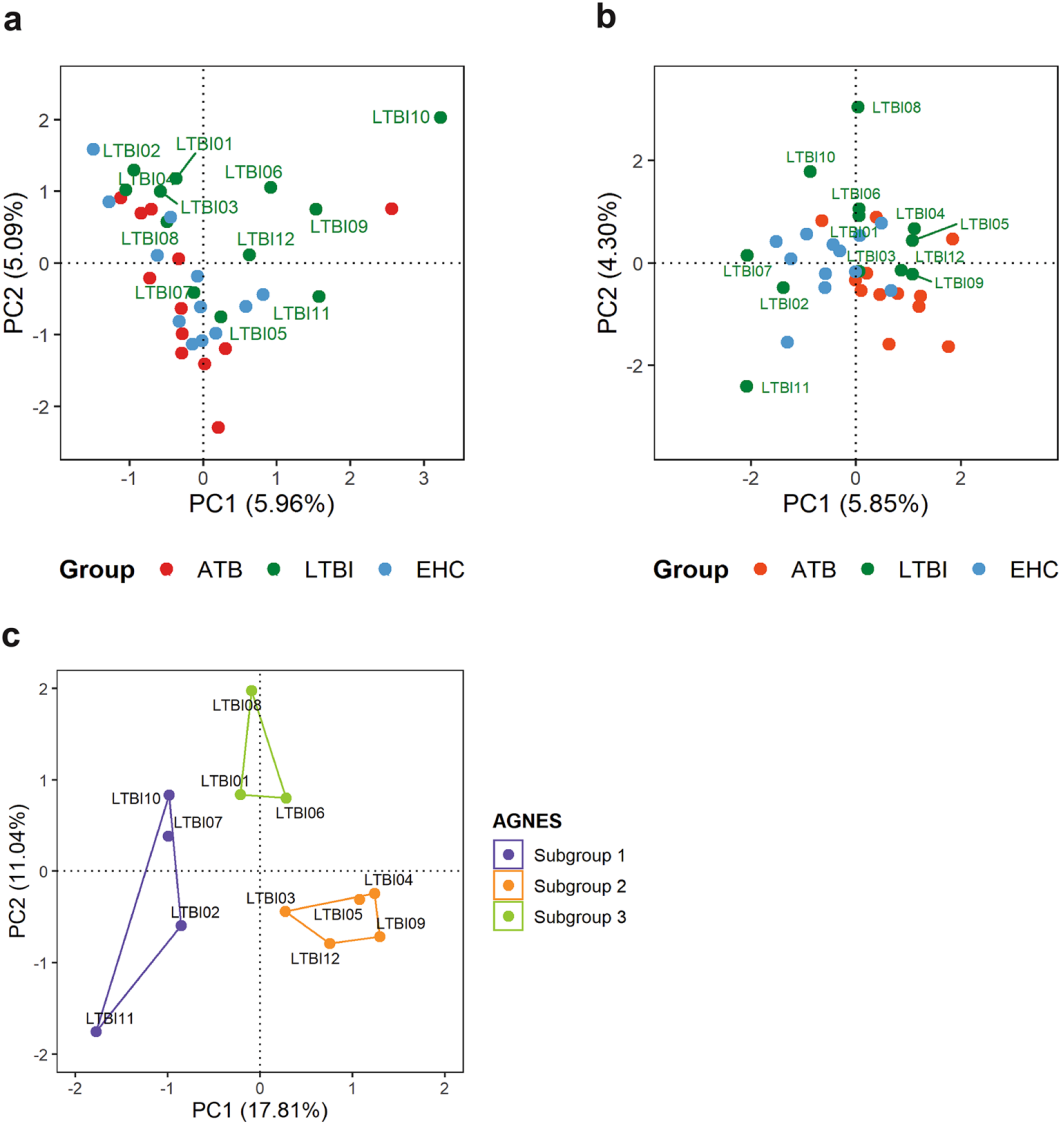
Principal component analysis of peptide-specific antibody responses and identification of LTBI subgroups. Individual factor maps (PCA) of IgG (**a**) and IgA responses (**b**), characterised by 11,766 and 7809 peptides, respectively, demonstrated distribution of individual samples (n = 36). Greater distances between ATB, LTBI, and EHC groups and within LTBI samples were notable in IgA dataset. LTBI subgroups were suggested by differing responses to the drPeptides and later identified by PCA and AGNES using IgA signals against all 16,730 peptides in the library (**c**).

Epitopes of dormancy-associated *M. tb* antigens that were recognized by circulating IgG and IgA antibodies among LTBI individuals were identified by high content peptide microarray. Derived from 52 dormancy-associated proteins, the 16,730 overlapping peptide library were tested with plasma of ATB, LTBI, and EHC groups (n = 12 from each group). The demographic characteristics of participants are summarized in Table 1. By means of median filtering, IgG and IgA datasets specific responses to 11,766 and 7809 peptides, respectively, were included for principal component analysis (PCA) for tendency grouping. Antibody profiling represented by PCA (Fig. 1) did not clearly differentiate ATB, LTBI, and EHC group. Nevertheless, greater distances between individual samples and groups were noted from IgA profile (Fig. 1b), in comparison to IgG (Fig. 1a). The spatial distributions of IgA response in LTBI also tended to form multiple clusters.

**Table 1:** Characteristics of subjects for peptide microarray screening.

Category	Endemic healthy control (EHC)	Latent tuberculosis infection (LTBI)	Active tuberculosis (ATB)
Total number of subjects (n)	12	12	12
Median age (range)	36.25 (24–52)	38.5 (26–52)	37.5 (20–54)
Gender			
Male, n (%)	9 (75%)	7 (58.33%)	11 (91.67%)
Female, n (%)	3 (25%)	5 (41.67%)	1 (8.33%)
QuantiFERON TB gold-in-tube			
Positive, n (%)	–	12 (100%)	12 (100%)
Negative, n (%)	12(100%)	–	–
Indeterminate, n (%)	–	–	–

These clusters were categorized based on agglomerative hierarchical clustering (AGNES) of original 16,730 peptide-specific IgA signals. The three LTBI subgroups, namely LTBIs1 (n = 4), LTBIs2 (n = 3), and LTBIs3 (n = 5), were graphically distinct from each other and were not associated with position on the microarray chips (Fig. 1c). Therefore, IgA responses were likely to be more dispersed and offered more promising for subsequent epitope identification by differential recognition analysis.

### Immunoreactivity of IgA against epitopes of dormancy-associated *M. tb* proteins in LTBI individuals.

**Figure 2:**
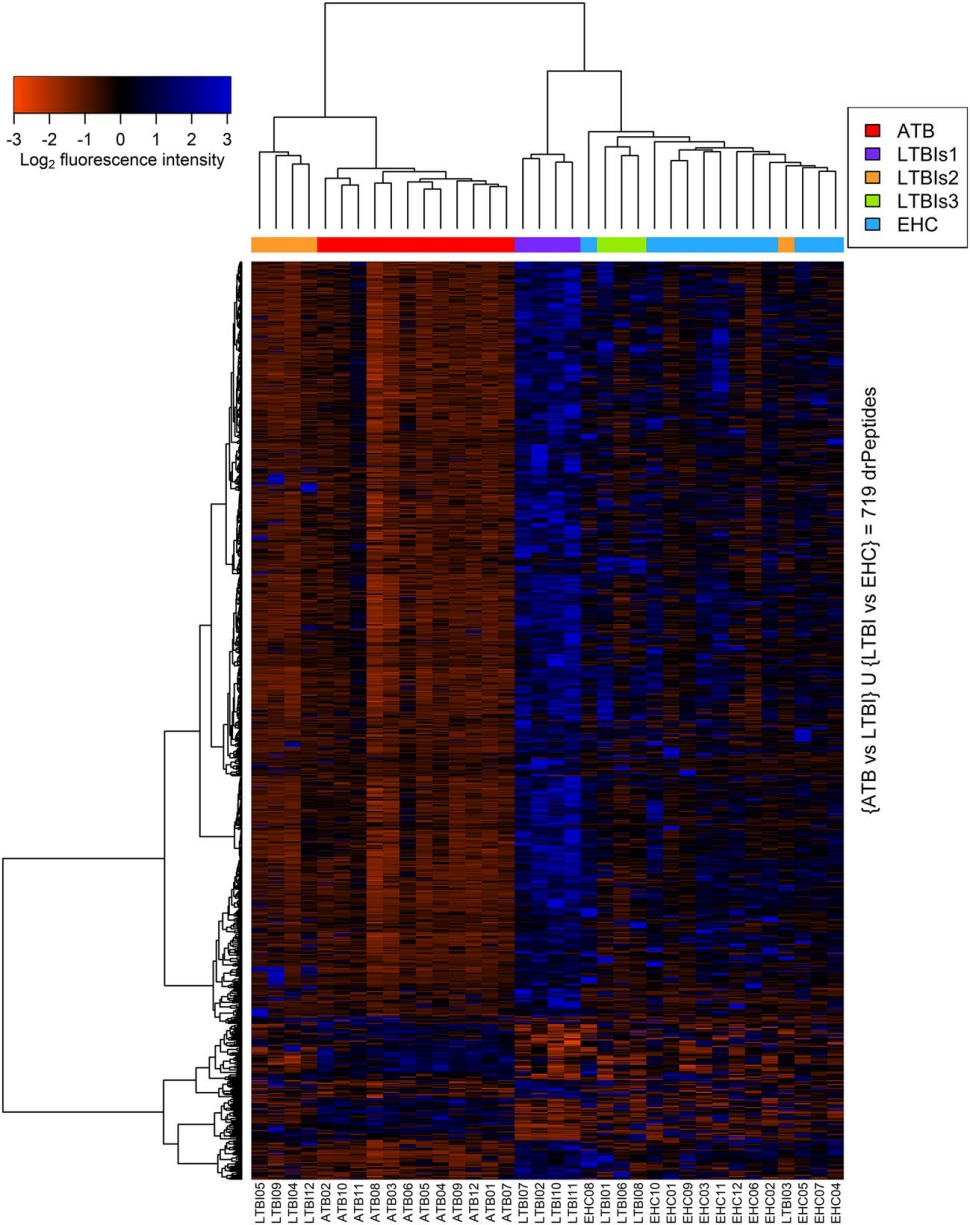
Heatmap and cluster analysis of IgA reactivity against 719 non-redundant drPeptides. IgA signals specific to 719 non-redundant peptides (horizontal axis), which had been identified from ATB v. LTBI (n = 715) and LTBI v. EHC (n = 8), are visualized across all 36 samples (vertical axis). The heatmap was generated using gplots package (heatmap.2) in the R/Bioconductor environment (https://cran.r-project.org/package=gplots). The level of IgA responses was represented by z-score of log_2_ normalised fluorescence intensity, in which 0 indicates the mean across 36 samples (black); positive (blue) and negative (red) values indicate standard deviation above and below the mean, respectively.

Differential recognition analysis was conducted by using *limma* package (R/Bioconductor) to identify differentially recognized peptides (drPeptides). The level of peptide-specific IgG was not significantly different between ATB, LTBI, and EHC. On the other hand, peptide-specific IgA differentially recognized 54 peptides in comparison between ATB v. LTB. Of these, 9/54 peptides were under-recognised by ATB, producing lower IgA responses than LTBI (Supplementary Table 2). We further characterize within-group variation of the LTBI subgroups, whose IgA responses were individually compared with ATB and EHC by differential recognition analysis. Combining the results from 3 LTBI subgroups (LTBs1 + LTBs2 + LTBIs3), 715 non-redundant drPeptides were identified between ATB v. LTBI (Supplementary Table 4–6)and 8 drPeptides between LTBI v. EHC (Supplementary Table 7–9). Note that some peptides were shared across comparisons. The heatmap visualizing IgA responses against 719 non-redundant drPeptides (ATB v. LTBI and LTBI v. EHC) suggested that LTBI individuals elicited diverse responses (Fig. 2). LTBIs1 contributed to the majority of additional drPeptides, producing the highest magnitude of IgA signals against 620 peptides than ATB. Of these, EHC also showed significantly higher IgA responses to 158 peptides than ATB. LTBIs2 and LTBIs3 demonstrated lower IgA to these set of drPeptides. Nevertheless, the hierarchical clustering shown on the heatmap clearly distinguished IgA responses of ATB from certain LTBI subgroups and EHC. These findings suggested that specific IgA responses against dormancy-associated antigens may be useful in characterizing the *M. tb* infection spectrum.

**Figure 3:**
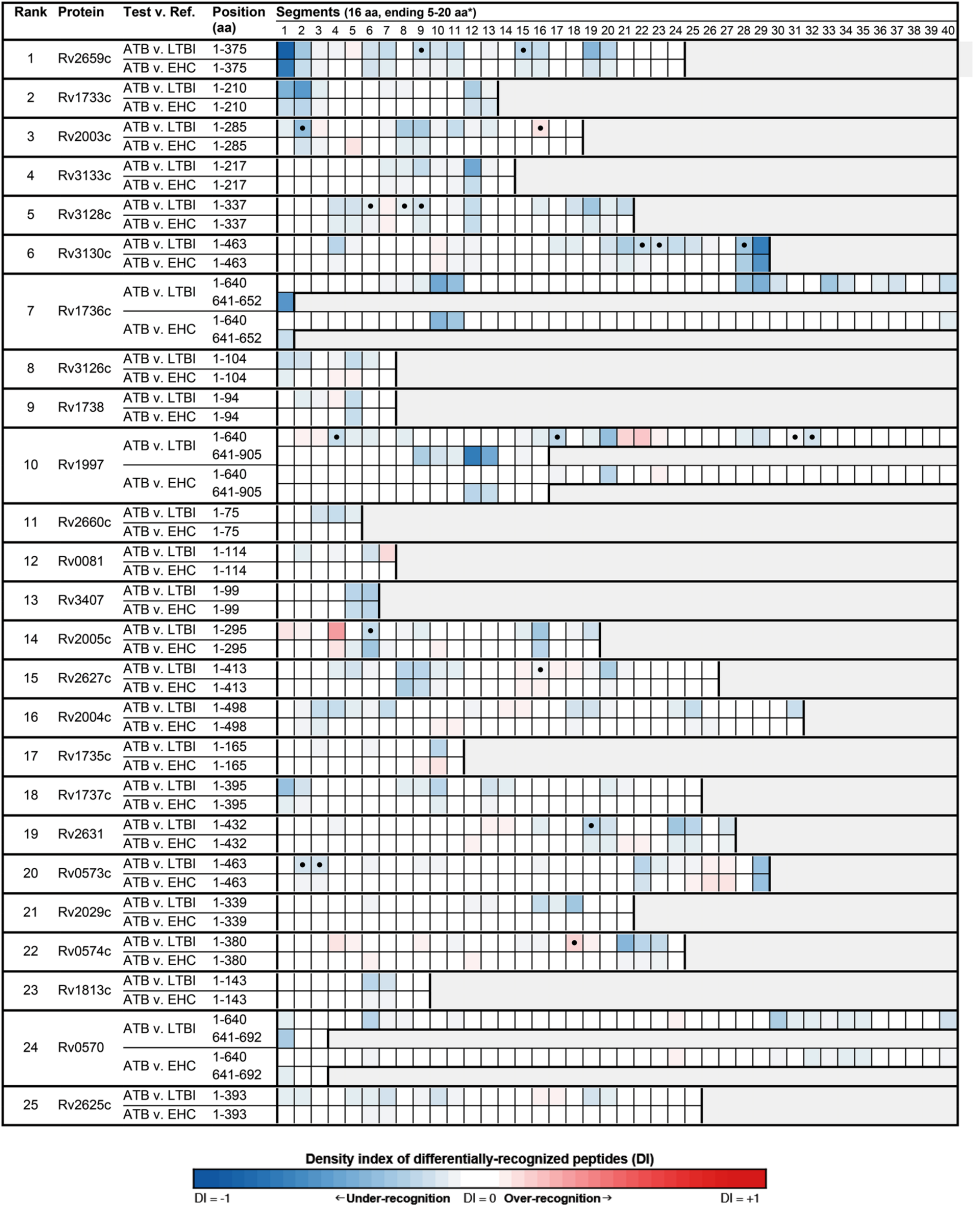
Sequential epitope diagram showing hotspots of 25 top differential IgA recognition in ATB v. LTBI. The aa sequences of the proteins are graphically presented in columns (marked by numbers 1–40) of 16-aa segments. DI of differentially-recognized peptides where DI of − 1 (blue) and 1 (red) indicate at least 5 overlapping under-recognized and over-recognized aa, respectively. The absence of differential recognition between groups is marked by DI of 0 (white). The symbol (closed circle) indicates a co-localized segment of drPeptides identified from both ATB and at least two subgroups of LTBI.

Using the peptide microarray data, sequential epitope mapping was generated to identify differential recognition hotspots on the protein sequence and the immunoreactivity of IgA against the proteins. Each drPeptide identified between ATB v. LTBI and ATB v. EHC was aligned, if overlapped at least 5 aa, to the aa sequences of dormancy-associated proteins (Supplementary Fig. S1). Probability of IgA-binding sites on a specific 16-aa protein segment was represented by density index of the drPeptides (DI) (Fig. 3). This sequential epitope mapping also highlighted a broader span of immunogenic hotspots with stronger IgA reactivity in both LTBI and EHC groups.

Among 715 non-redundant drPeptides in comparison between ATB and LTBI, 636 peptides were under-recognized by ATB group. The percentage of these drPeptides producing higher IgA responses among LTBI individuals was subsequently used to determine the degree of immunoreactivity of each protein. The top 10 most immunoreactive proteins were Rv2659c, Rv1733c, Rv2003c, Rv3133c, Rv3128c, Rv3130c, Rv1736c, Rv3126c, Rv1738 and Rv1997 (Fig. 3). Therefore, these high immunoreactive proteins screened by the high-content peptide microarray were chosen to study the immunoreactivity in the larger scale.

### Plasma IgA and IgG antibody responses to recombinant proteins Rv2659c, Rv1738 and ESAT-6 among HHC, LTBI and ATB

To validate the result from the screening phase, we selected Rv2659c and Rv1738 for evaluation of immunoreactivity in a larger study group. Selection of the dormancy-associated antigens was based on the first rank in the antibody-profile screening (Rv2659c), and their ability to trigger innate immune response (Rv2659c and Rv1738)^20^. ESAT-6, the active phase antigen, was also included in the study. Recombinant Rv2659c, Rv1738 and ESAT-6 proteins were constructed with molecular weights of 43 kDa, 13 kDa and 11 kDa, respectively, (Supplementary Fig. S3). We then performed an indirect ELISA to investigate whether IgG and IgA from the three study groups (61 HHC, 76 LTB, and 82 ATB) react against these recombinant proteins. The demographic characteristics of these subjects are summarized in Table 2.

**Table 2:** Characteristics of subjects for humoral immune response study.

Category	Household contact (HHC)	Latent tuberculosis infection (LTBI)	Active tuberculosis (ATB)
Total number of subjects (n)	61	76	82
Median age (range)	39 (18–85)	46 (18–80)	51 (18–86)
Gender			
Male, n (%)	17 (27.87%)	27 (35.53%)	60 (73.17%)
Female, n (%)	44 (72.13%)	49 (64.47%)	22 (26.83%)
QuantiFERON TB gold-in-tube			
Positive, n (%)	–	76 (100%)	75 (91.46%)
Negative, n (%)	61 (100%)	–	4 (4.9%)
Indeterminate, n (%)	–	–	3 (3.65%)

**Figure 4:**
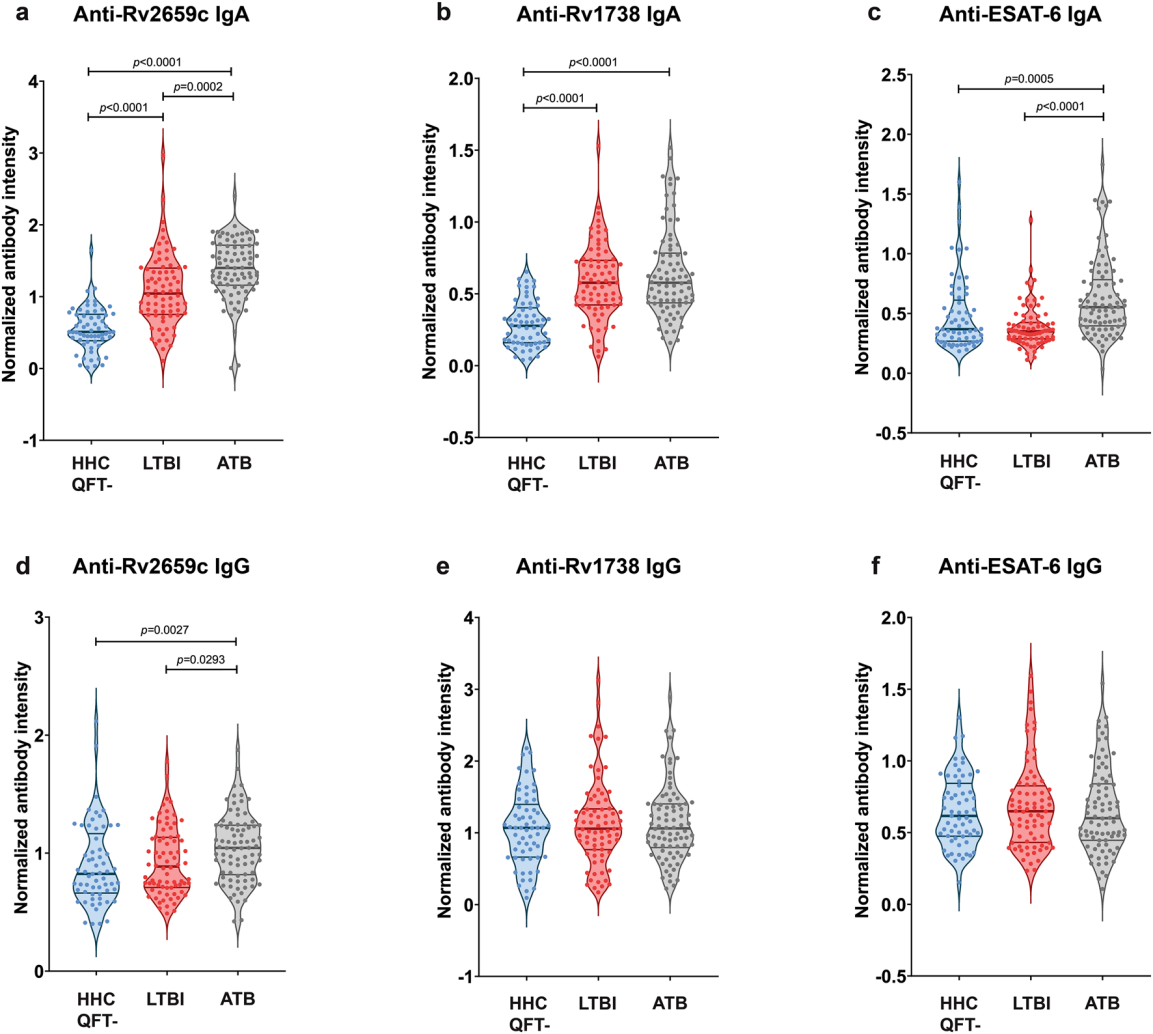
Levels of IgG and IgA antibody responses against proteins Rv2659c, Rv1738 and ESAT-6 by study groups. Quantitation of plasma antibodies in HHC (n = 61), LTBI (n = 76) and ATB (n = 82) subjects against proteins Rv2659c (**a**, **d**), Rv1738 (**b**, **e**) and ESAT-6 (**c**, **f**) was performed by indirect ELISA. Individual antibody-reactivity was represented as the normalized antibody intensity, calculated by the OD values of each sample multiplied by the normalized factor (OD of serum control divided by mean OD values in the same plate). Data are demonstrated in violin plot with median and interquartile range. One-way ANOVA Kruskal–Wallis test following Dunn’s multiple comparison was used for statistical analyses. A *p*-value < 0.05 was considered statistically significant.

The results showed that Rv2659c-specific IgA antibody level in LTBI were lower than those of ATB (*p* < 0.05), while the level of Rv1738-specific IgA antibody in LTBI were comparable to ATB (Fig. 4a,b). Interestingly, both Rv2659c-and Rv1738-specific IgA antibody levels in LTBI were higher than those of HHC (*p* < 0.05) (Fig. 4a,b). Moreover, the ESAT-6-specific IgA levels in ATB were remarkably greater than those of HHC (*p* < 0.05) and LTBI (*p* < 0.05), while there was no difference between LTBI and HHC (Fig. 4c). Regarding IgG responses, Rv2659c-specific IgG antibody levels in ATB were substantially greater than those in LTBI and HHC groups (*p* < 0.05 for each) (Fig. 4d). IgG antibody levels against Rv1738 and ESAT-6 did not significantly differ among the groups. (Fig. 4e,f). These results indicated that *M. tb*-infected individuals in the high TB burden country, Thailand, had detectable levels of both specific IgA and IgG antibodies against Rv2659c, Rv1738 and ESAT-6. Interestingly, the level of IgA from *M. tb*-infected individuals was significantly higher than those of household contacts.

### Specific CD4^+^ and CD8^+^ T cells to recombinant Rv2659c, Rv1738 and ESAT-6 proteins in HHC, LTBI and ATB

T cell-mediated immune response is essential for controlling *M. tb*. We, therefore, performed an intracellular cytokine staining by flow cytometry to detect specific T cell recognition against recombinant Rv2659c, Rv1738 and ESAT-6 proteins in PBMCs from the three study groups (30 HHC, 12 LTBI and 17 ATB). The demographic profiles of subjects are summarized in Table 3.

**Table 3:** Characteristics of subjects for cell-mediated immune response study.

Category	Household contact (HHC)	Latent tuberculosis infection (LTBI)	Active tuberculosis (ATB)
Total number of subjects (n)	30	12	17
Median age (range)	37 (18–85)	50 (27–75)	46 (19–84)
Gender			
Male, n (%)	16 (53%)	6 (50%)	9 (53%)
Female, n (%)	14 (47%)	6 (50%)	8 (47%)
QuantiFERON TB Gold-in-Tube			
Positive, n (%)	–	12 (100%)	N/A
Negative, n (%)	30(100%)	–	N/A
Indeterminate, n (%)	–	–	N/A

**Figure 5:**
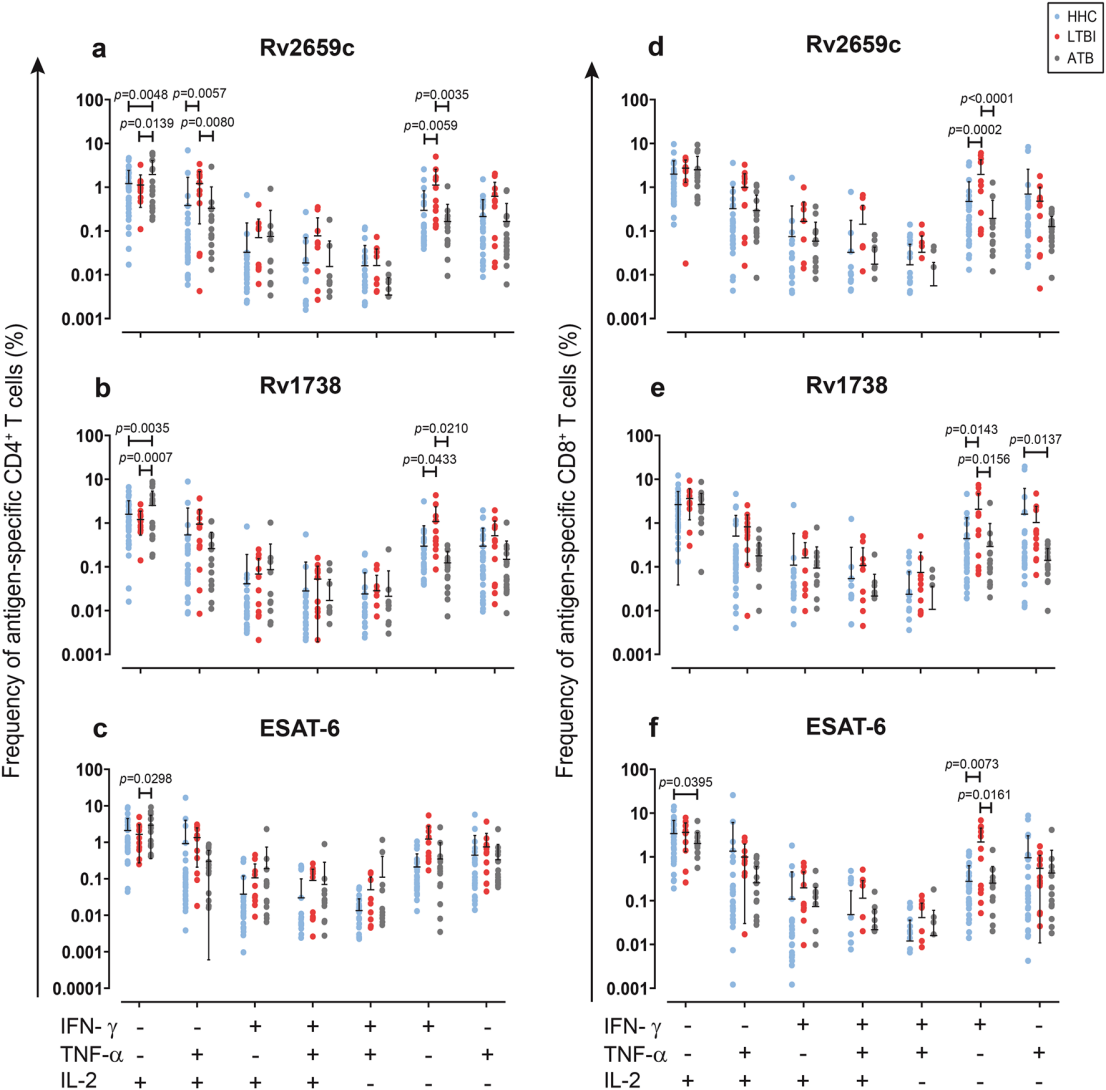
Intracellular cytokines specific CD4^+^ and CD8^+^ T cells upon stimulation with recombinant Rv2659c, Rv1738 and ESAT-6 proteins in HHC, LTBI and ATB subjects. PBMCs were stimulated with proteins Rv2659c (**a**, **d**), Rv1738 (**b**, **e**) or ESAT-6 (**c**, **f**) for 4 days. After stimulation, cells were harvested and stained for cell surface markers and intracellular cytokines as described in Materials and Methods. All possible combinations of the different functions are depicted on the X axis, whereas frequency of cytokine producing CD4^+^ and CD8^+^ T cells is depicted on the Y axis. Data were presented as mean ± SD. Two-way ANOVA following Sidak’s multiple comparisons test was performed; differences with *p* values < 0.05 were considered significant. Blue circles represent individuals with HHC (n = 30), red circles represent individuals with LTBI (n = 12) and grey circles represent individuals with ATB (n = 17).

The results showed that recombinant Rv2659c and Rv1738 induced a higher frequency of IFN-γ producing-CD4^+^ and CD8^+^ T cells in LTBI individuals compared to both ATB and HHC (*p* < 0.05) (Fig. 5a,b,d,e). In addition, LTBI subjects had a high frequency of multifunctional CD4^+^ T cell (IL-2 and TNF-α) upon stimulation with Rv2659c, but not with Rv1738 (Fig. 5a,b). However, a significant increase in IL-2 producing CD4^+^ T upon stimulation with ESAT-6- was observed only in the comparison between ATB and LTBI (Fig. 5c), while a significant increase in IFN-γ production from ESAT-6-stimulated CD8^+^ T cells was found in LTBI compared to both ATB and HHC groups (Fig. 5f). These results demonstrated that LTBI individuals from a high TB burden country elicited memory T cells to proteins Rv2659c and Rv1738 as well as ESAT-6.

## Discussion

Discovery of new antigens is crucial for TB vaccine development. Specifically, dormancy-associated *M. tb* antigens are potentially important targets for post-exposure vaccines in controlling TB reactivation. The immune responses to dormancy antigens are anticipated to control the dormant *M. tb* since most LTBI individuals do not develop ATB. The immune profiles retrieved from LTBI individuals in diverse geographic regions are distinct in their reactivity towards both dormancy- and active-phase antigens^19,21–23^. However, data on these immune responses in Thailand, a high TB burden country which uses BCG vaccination, is limited. In this study, our high-content peptide microarrays facilitated screening for, and identification of, numerous linear B cell epitopes of dormancy-associated *M. tb* antigens. Preferential IgA recognition against these epitopes was found in the LTBI group, though there was individual variation at the epitope level. Therefore, a summation of epitope-specific signals from each total protein was used to determine and rank IgA immunoreactivity scores. Interestingly, many proteins were identified to be in the top 10 rank have been reported as immunogenic in humans and mice. For example, studies from China and Japan demonstrated that Rv2659c, Rv3133c, Rv3130c and Rv1738 induce stronger T cell responses in LTBI than ATB individuals^24–26^. In addition, the multi-stage subunit antigens which included Rv1738 and ESAT-6 show promising therapeutic effects in a mouse model^26^. This indicated that our peptide microarray was able to reveal immunoreactive antigens of interest in a Thai population, and analyses showed them to be similar to those previously reported from other regions. We are aware of a small sample size, with 12 plasma samples per group, our data may not represent sufficient variability of target population (e.g. age, sex, BMI, high-risk and low-risk areas of residency, etc.). In our view, larger sample size is important to achieve a reliable power of discrimination by using ROC analysis.

To validate these findings, the immunological characteristics of recombinant proteins Rv2659c and Rv1738 were further evaluated in a larger study group. Proteins were selected based on their being first rank in the antibody-profile screening (Rv2659c) and their ability to trigger innate immune responses (Rv2659c and Rv1738)^20^. The presence of circulating IgG against selected dormancy antigens was determined in both LTBI and ATB subjects by indirect ELISA, eliciting the consistent results to the peptide array data. The IgG levels against Rv1738 exhibited no significant difference, while Rv2659c-specific IgG levels in ATB were markedly higher than those in LTBI. However, these results contrasted with previous studies that demonstrated no notable differences in Rv2659c-specific IgG in LTBI and ATB groups from China, a region with a similar high TB burden^24^. Moreover, IgG responses against Rv1738 in ATB were higher than those of LTBI individuals in Japan^25^. These differences may be due to host genetic polymorphism, bacterial prevalence, genetic diversity and dynamic distributions of *M. tb*^27^.

Besides IgG, circulating IgA levels were also evaluated in both LTBI and ATB groups, with no remarkable differences found between them. The results were not consistent with the peptide microarray data where the magnitude of IgA from LTBI subjects were higher than from ATB. This might be due to the small number of samples during antibody-immunoreactivity screening and the difference specific epitopes between peptides and recombinant proteins. Nevertheless, our previous report showed that circulating IgA memory B cells specific to Rv2659c were higher in LTBI individuals compared to ATB^28^. The discordance might be explained in that antibody levels do not necessarily reflect memory B cells. Others have reported finding no correlation between the frequency of circulating antigen-specific IgG memory B cells and the serum titers of antigen-specific IgG antibody^29,30^.

Apart from antibody, T cell and IFN-γ responses, the pivotal players in controlling *M. tb*, were also investigated in this study. The proportion of IFN-γ-producing CD4^+^ T cells induced by Rv2659c and Rv1738 were higher in LTBI than ATB individuals. These findings were concordant with previous reports from South Africa and China^26,31^. Importantly, the protective role of multifunctional T cells (T cells producing the combination of IFN-γ, IL-2, and/or TNF-α) has been raised as the crucial immune components against TB. For example, the proportion of IL-2 and IFN-γ-producing ESAT-6-specific CD4^+^ T cells and, IFN-γ- and TNF-α-producing Rv2029c-specific CD4^+^ T cells, are higher in LTBI than ATB individuals and associated with protective immunity to TB^15,32^. However, our study found that LTBI individuals exhibited proportions of antigen-specific multifunctional (IFN-γ^+^IL-2^+^ or IFN-γ^+^ TNF-α^+^) CD4^+^ T cells not greater than those in HHC and ATB subjects, however, the proportion of TNF-α and IL-2 producing Rv2659c-specific CD4^+^ T cells was higher than that of the other groups. Our results highlighted the combination of TNF-α and IL-2-producing T cells found in the LTBI group. Considering the role of IFN-γ in enhancement of macrophage function, IL-2 in T cell proliferation and TNF-α in the maintenance of granuloma structure, the presence of these particular functional T cell profiles in LTBI individuals may be necessary to the maintenance of granulomas and control of dormant *M. tb*.

Taken together, our report implies that the magnitude of adaptive immune responses to Rv2659c and Rv1738 in LTBI individuals in a high TB burden area that are comparable or higher than those in ATB might contribute to natural protection against dormant *M. tb* and be potential targets for a multi-stage vaccine. Further studies should assess their combination with active-phase antigens and evaluate their immunogenicity in the animal models.

## Materials and methods

### Ethical approval

The consent form and process, specimen collection, and the experimental protocol were reviewed and approved by the Central Institutional Review Board, Mahidol University, Thailand (No. MU-CIRB 2016/041.2803). All experiments were conducted in accordance with relevant guidelines and regulations. Informed consent was obtained from all subjects.

### Study subjects and samples

Heparinized blood samples from individuals with active tuberculosis (ATB) (n = 111), latent tuberculosis infection (LTBI) (n = 100) and household contact (HHC) (n = 91) included in this study were recruited from Chiangrai Prachanukroh Hospital, Chiangrai Province, Thailand between August 2017 till March 2022. The active TB patients were diagnosed following the WHO guidelines^33^, including TB-specific clinical symptoms, chest radiograph abnormalities, AFB sputum smears positive and/or bacterial culture positive. The asymptomatic close contacts of ATB patients were classified as having latent TB infection (LTBI) if the QuantiFERON TB Gold-in-Tube (QFT) (Cellestis, Carnegie, Australia) was positive, and classified as household contacts (HHC) if the QFT was negative. The endemic healthy controls (EHC) (n = 12) were recruited from healthy persons living in Bangkok without TB and having negative QFTs in conjunction with normal chest X-ray. Diagram of study design was shown in Supplementary Fig. S2.

### High-content peptide microarray screening

An overlapping 16-mer peptide library was constructed using amino acid sequences of *M. tb* H37Rv. The peptide library represented 52 dormancy-associated *M. tb* proteins, consisting of 47 entries regulated by DosR regulon, two regulated by starvation stimulon, and one each from *Rv2026c*, *Rv2027c (dosT)*, and *Rv3407* (Rv2628c was excluded due to manufacturing error) (Supplementary Table S1). The two million-spot capacity of high-content peptide microarray chips (Roche NimbleGen, USA) was allocated into 12 subarrays, in which 8 replicates of the peptide library were printed in 8 blocks, resulting in 133,840 data points per sample. Plasma samples diluted 1:100 in binding buffer (0.01 M Tris–Cl, pH 7.4, 1% alkali-soluble casein, 0.05% Tween-20) were applied to probe microarray chips overnight at 4℃ and subsequently submerged in washing buffer (TBS, 0.05% Tween-20) for 10 min (3 times). Alexa Fluor® 647-conjugated goat anti-human IgG and fluorescein-conjugated AffiniPure Goat Anti-Human IgA (Jackson ImmunoResearch) diluted in binding buffer (TBS, 1% alkali-soluble casein, 0.05% Tween-20) to a final concentration of 0.1 ng/µl were used to detect IgG/IgA bindings by 3 h incubation at RT. Then, microarray chips were washed with washing buffer for 10 min (3 times) and rinsed with reagent-grade water. Fluorescence signals were detected by an MS200 Microarray Scanner (Roche NimbleGen, USA) at 635 nm for anti-IgG and 532 nm for anti-IgA at 2 µm resolution and 25% gain.

### Peptide microarray data analysis

Fluorescence signals from the peptide microarray scans were digitized by NimbleScan Software version 2.5 (Roche NimbleGen, USA). Background noise was corrected by robust multiarray average (RMA) and within-array spatial correction by 2D-Loess regression. The data were quantile normalized and log_2_ transformed, and the median antibody responses against 16,730 peptides were determined from 8 within-array replicates. Fold changes of peptide-specific signals were calculated against the respective median across all samples. Peptides that displayed ≥ 2.5-fold increase or decrease in at least 10% of samples were retained for further analysis. These included 11,766 and 7809 peptides from IgG and IgA datasets, respectively. Principal component analysis (PCA) is an unsupervised method used to explore multivariate datasets, including grouping tendency and pattern. PCA in conjunction with agglomerative hierarchical clustering (AGNES) were used to identify subgroups within LTBI samples. AGNES was calculated by Euclidean distance and Ward’s minimum variance method.

The IgG/IgA differential recognition was determined by linear model for microarray data (limma) package, followed by Benjamini–Hochberg false discovery rate correction at significance level (p-value) < 5%. Pairwise comparisons of peptide-specific binding among ATB, LTBI, and EHC samples were performed to identify differentially recognized peptides (drPeptides), which were then aligned with the original amino acid chains of the proteins. By dividing the amino acid chains into frame of consideration of 16-aa segments, we counted drPeptides that overlapped at least 5 aa residues as part of the segments. Probability of differential recognition hotspot was defined in this study as density index of drPeptides (DI). The number of drPeptides appertaining to each segment was divided by the number of all possible peptides in the library that made up the segment (i.e. overlapped at least 5 aa). The value of DI ranges from − 1 to 1, where − 1 indicates hotspot of under-recognition (lower antibody response), 0 no differential antibody recognition, and 1 hotspot of over-recognition (higher antibody response).

### Construction of recombinant proteins and enzyme-linked immunosorbent assay

Recombinant proteins were constructed as previously described^28^ (Supplementary Fig. S3). The specific IgG and IgA antibodies against recombinant Rv2659c, Rv1738 and ESAT-6 proteins were investigated using an indirect ELISA. Ninety-six-well microplates (Corning, NY, USA) were coated with 25 µl of Rv2659c, Rv1738 and ESAT-6 proteins at 20 ng/µl in HBS buffer (HBS containing 1 mM MnCl_2_) and incubated overnight at 4 °C. After washing, plates were blocked with blocking buffer (5% BSA in HBS pH 7.6) for 2 h at RT. The 25 µl diluted plasma samples (1:200 and 1:100 dilution for IgG and IgA detection, respectively) were added and incubated for 1 h at RT. All plasma samples and controls were run in duplicate on the same plate. After a washing step, plates were incubated for 1 h at RT with 25 µl with one of the following secondary antibodies: pre-adsorbed goat anti-human IgG H + L-horseradish peroxidase (Abcam, UK) or peroxidase-labelled anti-human IgA (KPL, USA). Plates were washed and enzyme activity was detected by adding 25 μl/well TMB substrate (Merck KGaA, Sigma-Aldrich, USA) for 15 min. The reaction was stopped with 25 μl/well 1N HCl and the absorbance was read at OD 450 nm.

### Intracellular cytokine staining by flow cytometry

Peripheral blood mononuclear cells (PBMCs) were isolated by Lymphoprep (Stemcell, Canada). PBMCs (5 × 10^5^ cells/well) were seeded in 96-well cell culture plate (Corning, NY, USA) and stimulated for 4 days with 15 µg/ml of Rv1738, Rv2659c or ESAT-6 at 37 °C with 5% CO_2_. Phorbol 12-myristate 13-acetate (Sigma, USA) at 25 ng/ml and Ionomycin (Sigma, USA) at 1 µg/ml were used as positive controls. Non-stimulated cells were used as negative controls. Cells were incubated in the presence of 10 µg/ml Brefeldin A (Sigma, USA) for 4 h before the end of culture. The PBMCs were then harvested and stained with the following cell surface markers: Alexa fluor700 anti-CD3, PerCP-Cy5.5 anti-CD4, APC-Cy7 anti-CD8 (at 4 °C for 15 min). Subsequently, cells were fixed and permeabilized with a Cytofix/Cytoperm Kit (BD Biosciences, USA) and then stained with PE-Cy7 anti-IFN-γ, FITC anti-IL-2 and APC anti-TNF-α at 4 °C for 30 min. All antibodies were purchased from BioLegend, USA. Data were acquired using a FACS Canto II (BD Biosciences, USA) and were analysed by FlowJo software (Tree Star, USA).

### Statistical analysis

Microarray data analyses were performed in an R/Bioconductor environment, using *limma* and other supporting software packages. GraphPad Prism 8.0 (GraphPad Software, San Diego, CA) was used for all statistical analyses. The one-way analysis of variance (ANOVA) with Kruskal–Wallis test was used to compare the level of specific IgG and IgA antibodies across the study group. The 2-way ANOVA with Sidak’s multiple comparisons test was used to compare the difference of frequency of cytokine-producing T cells in response to each protein between study groups. Results with *p* < 0.05 were considered as statistically significant.

## Supplementary Information

Supplementary Figures.

Supplementary Tables.

## Data Availability

The data supporting the findings of this study are available from the corresponding author on reasonable request.
