# Supplementary material for: Peptide microarray-based identification of dormancy-associated Mycobacterium tuberculosis antigens inducing immune responses among latent tuberculosis infection individuals in Thailand
Source: Sci Rep. 2023 Apr 28;13:6978. doi: 10.1038/s41598-023-34307-4 (PMC10141872; doi:10.1038/s41598-023-34307-4)
Supplement: Supplementary file 1 — Supplementary Figures. [file 41598_2023_34307_MOESM1_ESM.pdf]

Supplementary information

Peptide microarray-based identification of dormancy-associated *Mycobacterium tuberculosis* antigens inducing immune responses among latent tuberculosis infection individuals in Thailand

Jariya Hanthamrongwit[1 π], Panicha Aruvornlop[1 π], Chutiphon Saelee[1], Nattiya Wanta[2], Passarun Poneksawat[2], Phyu Thwe Soe[1,3], Soe Paing Kyaw[4], Prasong Khaenam[5], Saradee Warit[6], Davide Valentini[7], Surakameth Mahasirimongkol[8], Panadda Dhepakson[8], Sakulrat Soonthornchartrawat[8], Patchanee Chootong[1], Chaniya Leepiyasakulchai[1 *]

Supplementary Fig.S1

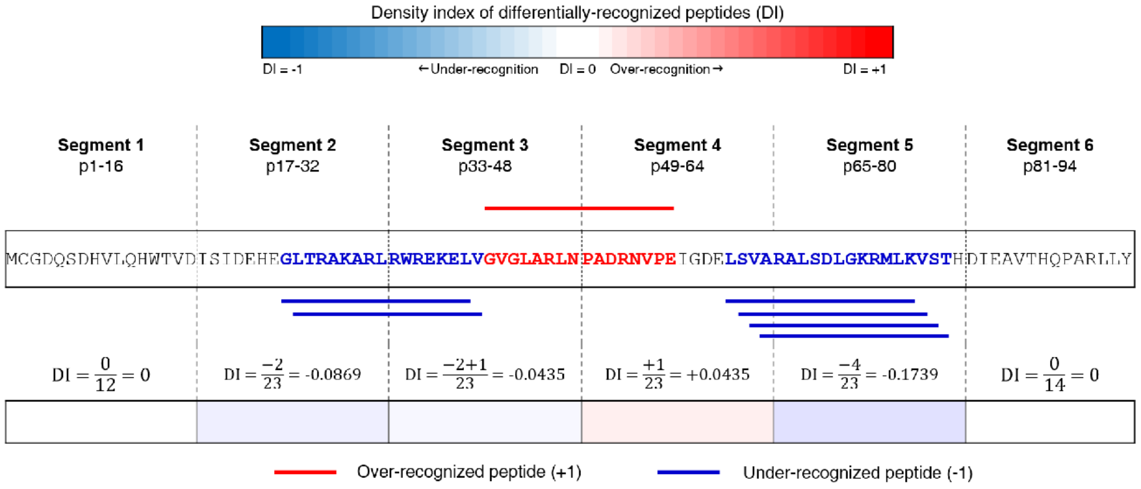

Supplementary Fig. S1. Schematic of sequential epitope mapping approach.

The aa sequence of a protein is represented by sequential segments, each of which is a consensus sequence of peptides (16-mer to 12-mer) that overlap each other by no less than 5 aa. The segments which contain aa residues of the drPeptides are highlighted by colours on the density index of differentially-recognized peptides (DI) scale, indicating the likelihood of an antibody-binding site (epitope) being found in the segment. By assigning the value of +1 to each over-recognized peptide (red) and -1 to each under-recognized peptide (blue), the DI is determined as the quotient between the sum value of all drPeptides in each segment (overlapping at least 5 aa) and the number of all possible peptides that constitute that segment.

Supplementary Fig. S2

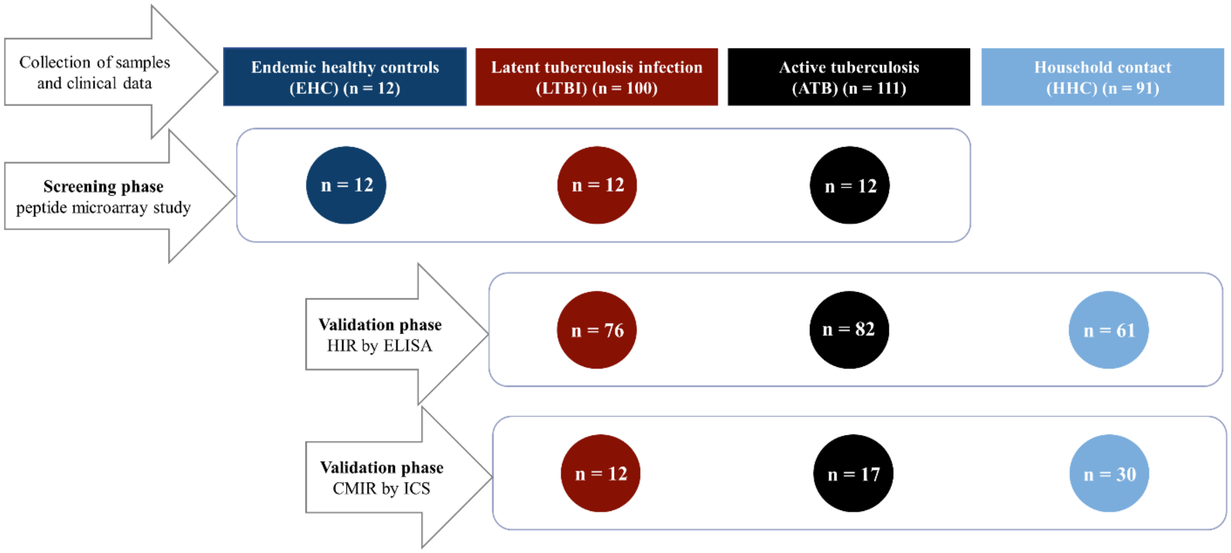

Supplementary Fig. S2. Schematic diagram of study design.

A total of 314 individuals were enrolled in the study. In screening phase, 36 individuals were recruited for peptide microarray study. In validation phase, plasma from 219 individuals were examined the level of antibody while PBMCs from 59 individuals were determined the proportion of antigen specific T cels.

Supplementary Fig. S3

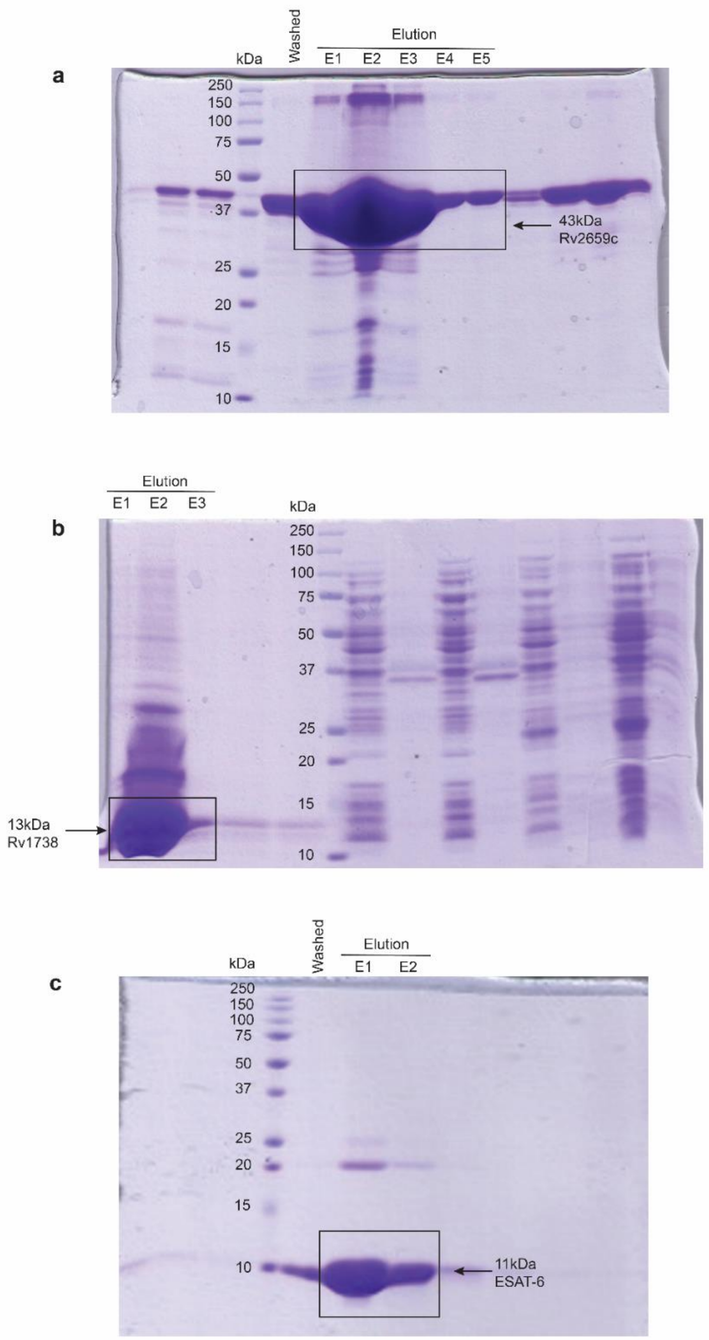

Supplementary Fig. S3. Expression and purification of recombinant Rv2659c, Rv1738 and ESAT-6 protein.

The recombinant plasmids pET24b-Rv2659c, pET28a-Rv1738 and pET24b-ESAT-6 were transformed into *E. coli* BL21 (DE3). The proteins were then induced with 0.5mM IPTG at 37°C for 3 hours (Rv2659c and ESAT-6) or overnight (Rv1738). Following protein extraction from bacterial cells, his-tagged proteins were purified by immobilized metal ion affinity chromatography using TALON. SDS PAGE gel images showing the elution fractions of recombinant Rv2659c (43kDa) (a), Rv1738 (13kDa) (b) and ESAT-6 (11kDa) (c) proteins using Coomassie blue staining.

Supplementary Table

Supplementary Table 1-9 are provided in excel file format.
